# Supplementary material for: Spatial N-glycan rearrangement on α5β1 integrin nucleates galectin-3 oligomers to determine endocytic fate
Source: Nat Commun. 2025 Oct 27;16:9461. doi: 10.1038/s41467-025-64523-7 (PMC12559291; doi:10.1038/s41467-025-64523-7)
Supplement: Supplementary file 1 — Supplementary Information [file 41467_2025_64523_MOESM1_ESM.pdf]

SUPPLEMENTAL INFORMATION FOR

Spatial N-glycan rearrangement on $\alpha_5\beta_1$ integrin nucleates galectin-3 oligomers to determine endocytic fate

Massiullah Shafaq-Zadah[1,2]†*, Estelle Dransart[1,2]†, Ilyes Hamitouche[1,2,3], Christian Wunder[1,2], Valérie Chambon[1], Cesar A. Valades-Cruz[1,4,5]‡, Ludovic Leconte[4,5], Nirod Kumar Sarangi[6], Jack Robinson[6], Siau-Kun Bai[1], Raju Regmi[7], Aurélie Di Cicco[7], Agnès Hovasse[8,9], Richard Bartels[3], Ulf J. Nilsson[10], Sarah Cianférani-Sanglier[8,9], Hakon Leffler[11], Tia E. Keyes[6], Daniel Lévy[7], Stefan Raunser[12], Daniel Roderer[3]*, and Ludger Johannes[1,2]*

Corresponding authors email: ludger.johannes@curie.fr, roderer@fmp-berlin.de, massiullah.shafaq-zadah@curie.fr

This pdf file includes:

Supplementary Figures 1 to 12 with captions
Supplementary Table 1

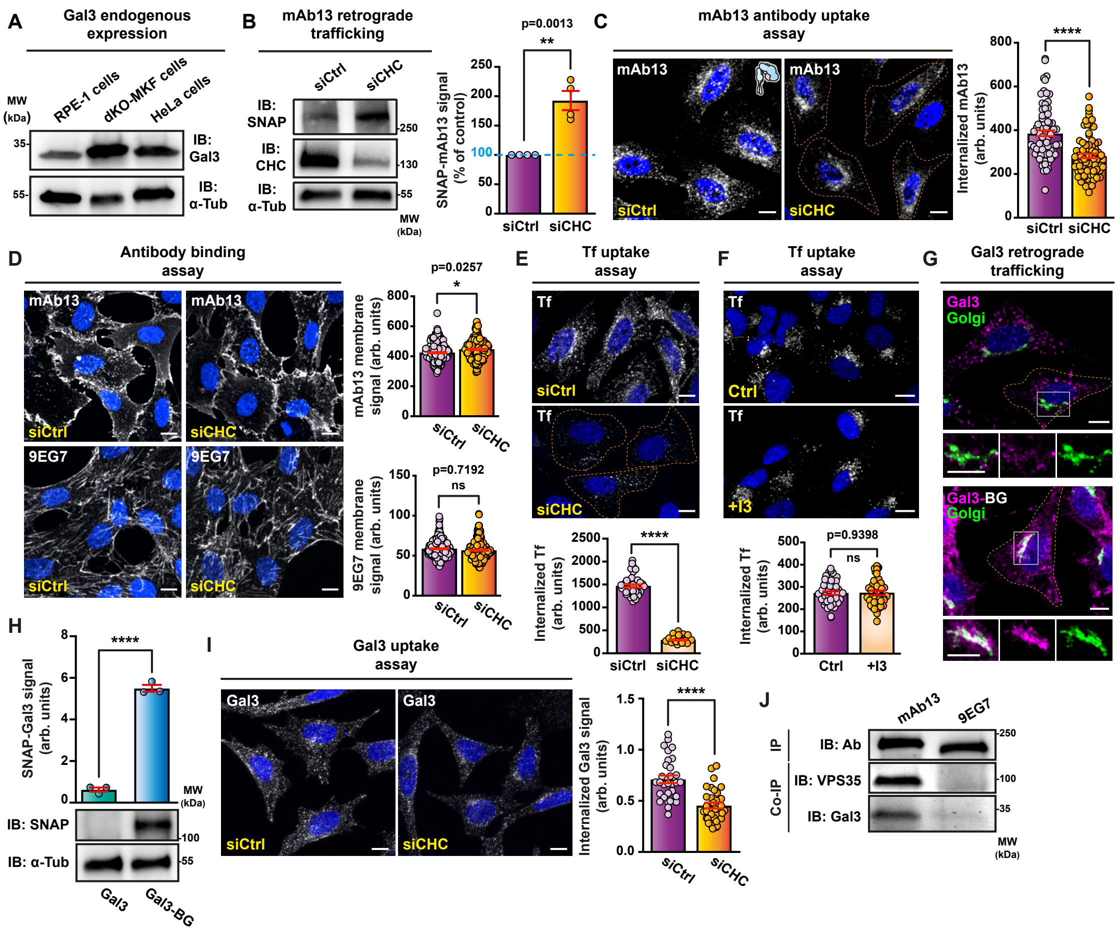

Supplementary Figure 1. Clathrin-independent retrograde trafficking of inactive bent-closed $\alpha_5\beta_1$ integrin depends on Gal3. (**A**) Endogenous expression of Gal3. Lysates from the indicated cell lines used in our study (RPE-1, dKO-MKF and HeLa) were analyzed by SDS-PAGE and immunoblotting for Gal3. (**B**) Continuous incubation of GalT-GFP-SNAP expressing HeLa cells that had continuously been incubated for 3 h at 37 °C with mAb13-BG, either in control condition (siCtrl) or after inhibition of clathrin expression (siCHC). mAb13-BG that reached the Golgi was quantified by immunoblotting (IB: SNAP) for the corresponding SNAP-mAb13 conjugates. Clathrin inhibition was assessed by immunoblotting (IB: CHC). α-tubulin was used for normalization (IB: α-Tub). n = 4 independent experiments. Means ± SEM, unpaired two-sided t-test; **P < 0.002. (**C**) Quantification by confocal microscopy of mAb13 uptake after continuous incubation for 10 min at 37 °C with HeLa cells (GalT-GFP-SNAP), either in control condition (siCtrl), or after inhibition of clathrin expression (siCHC). n = 80 (for siCtrl) and n = 79 (for siCHC) cells, representative of 3 independent experiments. Means ± SEM, unpaired two-sided t-test; ****P < 0.0001. Scale bars = 10 µm. Nuclei in blue. (**D**) Quantification by confocal microscopy of mAb13 or 9EG7 surface levels in HeLa GalT-GFP-SNAP-expressing cells (30 min incubation at 4 °C) in control condition (siCtrl) or after inhibition of clathrin expression (siCHC). n = 80 (for mAb13) and n = 100 (for 9EG7) cells, representative of 3 independent experiments. Means ± SEM, unpaired two-sided t-test; ns = P > 0.05, *P < 0.05. Scale bars = 10 µm. Nuclei in blue. (**E**) Quantification by confocal microscopy of transferrin (Tf) uptake after continuous incubation for 10 min at 37 °C in HeLa cells (GalT-GFP-SNAP), either in control condition (siCtrl), or after inhibition of clathrin expression (siCHC). n = 40 cells per condition, representative of 3 independent experiments. Means ± SEM, unpaired two-sided t-test; ****P < 0.0001. Scale bars = 10 µm. Nuclei in blue. (**F**) I3 effect on Tf uptake. Tf was continuously incubated for 10 min at 37 °C with RPE-1 cells

that were either untreated or pre-treated with the cell-impermeable Gal3 inhibitor I3. Signals coming from internalized Tf were quantified. n = 45 cells per condition, representative of 3 independent experiments. Means ± SEM, unpaired two-sided t-test; ns = P > 0.05. Scale bars = 10 µm. Nuclei in blue. (**G**) Gal3 trafficking to the Golgi. Continuous incubation of GalT-GFP-SNAP-expressing HeLa cells for 1 h at 37 °C with Cy3-labelled Gal3 (200 nM) that was also BG-coupled, or not. Note that Gal3-BG colocalized in perinuclear Golgi with GalT-GFP-SNAP. At least 3 independent experiments with similar results were conducted. Scale bars = 10 µm. Nuclei in blue. (**H**) Use of GFP-trap to pull down stably expressed Golgi-localized GalT-GFP-SNAP from HeLa cells that had continuously been incubated for 3 h at 37 °C with Gal3 or Gal3-BG. SNAP-Gal3 was quantified by immunoblotting (IB: SNAP). α-tubulin was used for normalization (IB: α-Tub). n = 3 independent experiments. Means ± SEM, unpaired two-sided t-test; ****P < 0.0001. (**I**) Quantification by confocal microscopy of Gal3 uptake (200 nM) after continuous incubation for 10 min at 37 °C with HeLa cells (GalT-GFP-SNAP), either in control condition (siCtrl), or after inhibition of clathrin expression (siCHC). n = 30 (for siCtrl) and n = 32 (for siCHC) cells, representative of 3 independent experiments. Means ± SEM, unpaired two-sided t-test; ****P < 0.0001. Scale bars = 10 µm. Nuclei in blue. (**J**) Co-immunoprecipitation experiment. Gal3 and mAb13 or 9EG7 antibodies were sequentially incubated for 30 min at 4 °C with RPE-1 cells. After washes, cells were shifted for 20 min to 37 °C, lysed, and antibodies immunoprecipitated (IP) overnight at 4 °C. Immunoblotting for Vps35 (IB: VPS35) and antibodies (IB: Ab) revealed that only inactive $\alpha_5\beta_1$ integrin (mAb13) co-immunoprecipitated Vps35 and Gal3.

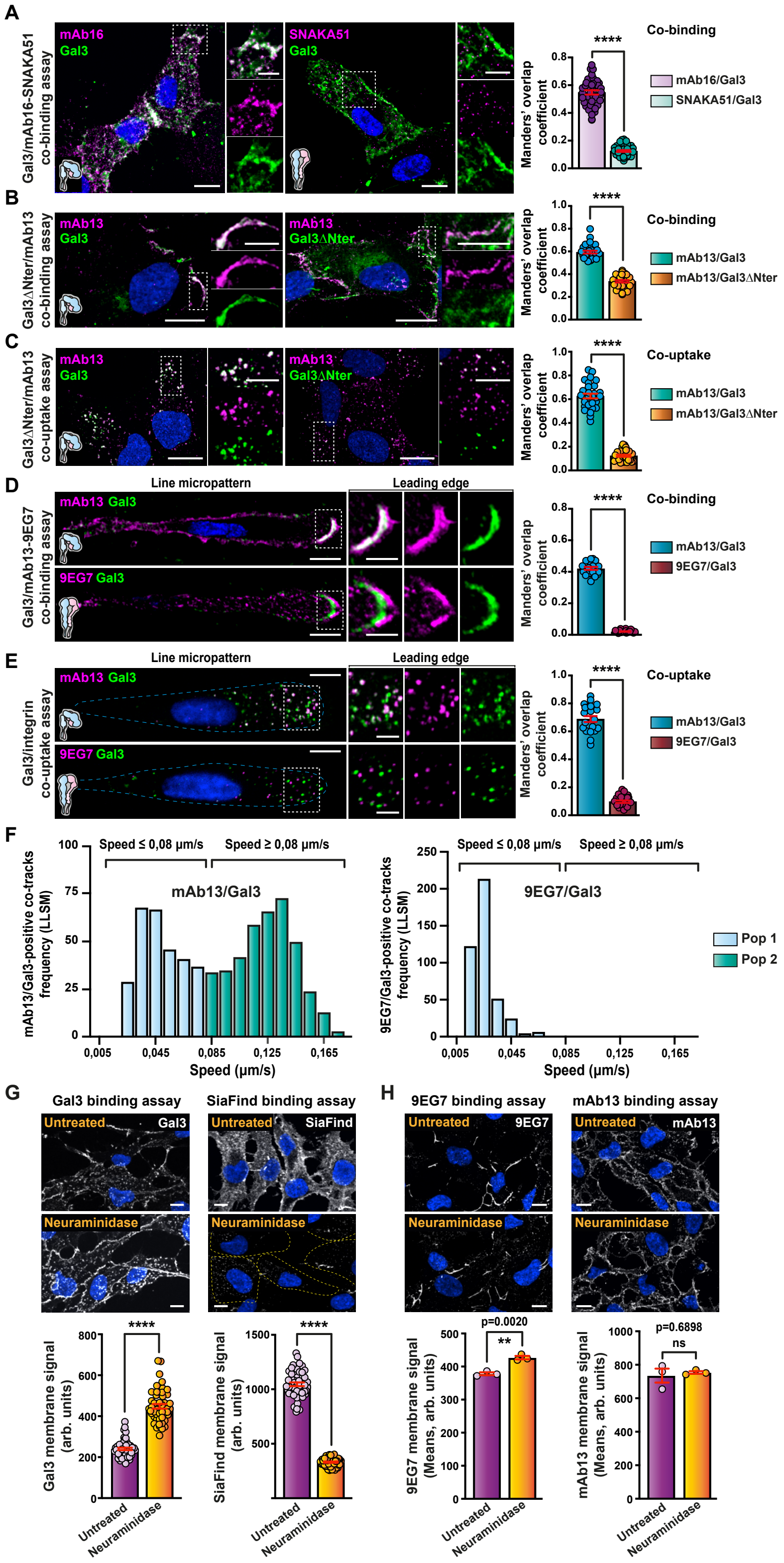

Supplementary Figure 2. Gal3 oligomerization capacity is required for its preferential binding to the inactive bent-closed conformational state of $\alpha_5\beta_1$ integrin. (**A**) Co-binding with α_5 integrin. RPE-1 cells were sequentially incubated at 4 °C with 200 nM of Gal3 and the conformation-specific α_5 integrin antibodies mAb16 (inactive) or SNAKA51 (active), and directly fixed. The overlap of fluorescence signals was quantified. n = 40 cells per condition were analyzed, representative of 3 independent experiments. Means ± SEM, unpaired two-sided t-test; ****$P < 0.0001$. Scale bars = 10 µm, and 5 µm in zoomed insets. Nuclei in blue. (**B**) Co-

binding with β_1 integrin. RPE-1 cells were sequentially incubated at 4 °C with 200 nM of Gal3 or Gal3ΔNter and mAb13 antibody, then directly fixed. The overlap of fluorescence signals was quantified. n = 30 cells per condition were analyzed, representative of 3 independent experiments. Means ± SEM, unpaired two-sided t-test; ****P < 0.0001. Scale bars = 10 µm, and 5 µm in zoomed insets. Nuclei in blue. (**C**) Co-uptake with β_1 integrin. RPE-1 cells were sequentially incubated at 4 °C with 200 nM of Gal3 or Gal3ΔNter and mAb13 antibody and then shifted for 10 min to 37 °C. The overlap of fluorescence signals was quantified. n = 30 cells per condition were analyzed, representative of 3 independent experiments. Means ± SEM, unpaired two-sided t-test; ****P < 0.0001. Scale bars = 10 µm, and 5 µm in zoomed insets. Nuclei in blue. (**D**) Same as in (B), using Gal3 and either mAb13 or 9EG7 antibody in RPE-1 cells that were seeded onto line micropatterns. Of note, Gal3 was massively distributed towards the leading edge and specifically overlapped with mAb13. The overlap of fluorescence signals was quantified. n = 30 cells per condition were analyzed, representative of 3 independent experiments. Means ± SEM, unpaired two-sided t-test; ****P < 0.0001. Scale bars = 10 µm, and 5 µm in zoomed insets. Nuclei in blue. (**E**) Same as in (C), using Gal3 and either mAb13 or 9EG7 antibody in RPE-1 cells that were seeded onto line micropatterns. The overlap of fluorescence signals was quantified. n = 30 cells per condition were analyzed, representative of 3 independent experiments. Means ± SEM, unpaired two-sided t-test; ****P < 0.0001. Scale bars = 10 µm, and 5 µm in zoomed insets. Nuclei in blue. (**F**) Lattice light sheet microscopy. Co-tracking of Cy3-labeled Gal3 with ATTO488-labeled mAb13 (left panel) or 9EG7 (right panel) antibody. Frequency distributions of Gal3-positive mAb13 or 9EG7 co-tracks in function of their velocity. Of note, the dynamic population (Pop 2) was totally absent for 9EG7/Gal3 co-tracks, indicating that 9EG7/Gal3-containing structures were largely immobile. (**G**) Effect of neuraminidase (sialidase) treatment on Gal3 binding to RPE-1 cells. RPE-1 cells were treated for 40 min at 37 °C with neuraminidase from *Arthrobacter ureafaciens*. Gal3 binding significantly increased on neuraminidase treated cells. Efficient removal of sialic acids from cell surface was assessed by incubating neuraminidase-treated or untreated cells for 30 min at 4 °C with the pan sialic acid-specific lectin SiaFind. In all conditions, cell surface fluorescence signals were quantified by confocal microscopy. n = 50 cells per condition, representative of 3 independent experiments. Means ± SEM, unpaired two-sided t-test; ****P < 0.0001. Scale bars = 10 µm. Nuclei in blue. (**H**) Effect of neuraminidase (sialidase) treatment on mAb13 and 9EG7 antibodies binding to RPE-1 cells. Experiment as in (G), showing no major effect of surface sialic acid removal on the binding levels of both conformer specific antibodies. n = 3 independent experiments with a total of 300 cells per condition. Means ± SEM, unpaired two-sided t-test; ns = P > 0.05, **P < 0.002. Scale bars = 10 µm. Nuclei in blue.

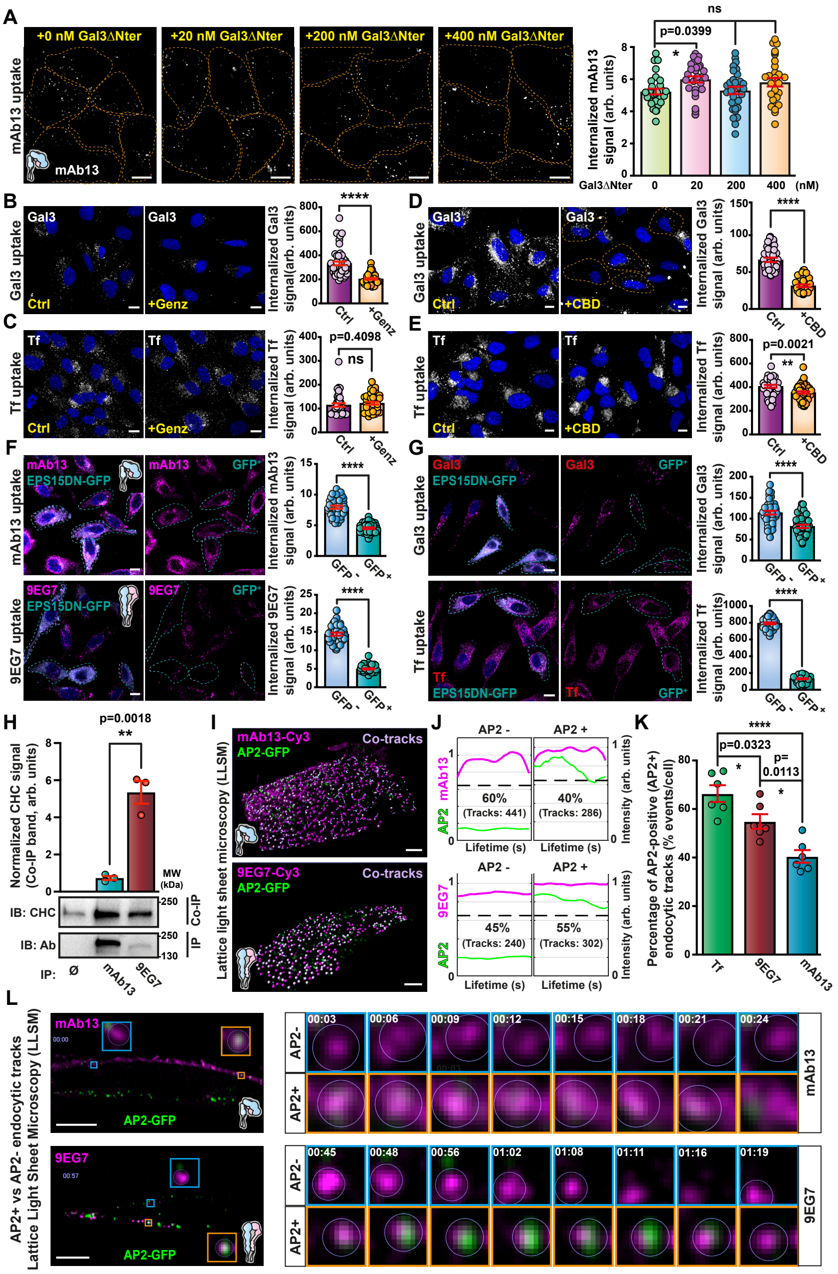

Supplementary Figure 3. Internalization of inactive bent-closed $\alpha_5\beta_1$ integrin relies on the GL-Lect driven endocytosis. (**A**) Gal3ΔNter effect on mAb13 uptake. mAb13 antibody was bound at 4 °C to RPE-1 cells, which were then incubated for 10 min at 37 °C in the absence or the presence of the indicated concentrations of exogenous Gal3ΔNter. Signals from internalized mAb13 were quantified. Dashed lines represent the contours of cells. n = 30 cells per condition, representative of 3 independent experiments. Means ± SEM, one-way ANOVA; ns = $P > 0.05$, *$P < 0.02$. Scale bar = 10 µm. (**B,C**) GSL depletion effect on endocytosis. Gal3 (200 nM) (B) or transferrin (Tf) (C) were continuously incubated for 5 min or 10 min, respectively, at 37 °C with RPE-1 cells that were pre-treated or not with the GSL synthesis inhibitor Genz. Signals from internalized Gal3 or Tf were quantified. n = 50 (for Gal3) and n = 40 (for Tf) cells were analyzed, representative of 3 independent experiments. Means ± SEM, unpaired two-sided t-test; ns = $P > 0.05$, ****$P < 0.0001$. Scale bars = 10 µm. Nuclei in blue. (**D,E**) Effect of the dynein inhibitor ciliobrevin D (CBD) on endocytosis. Gal3 (200 nM) (D) or transferrin (Tf) (E) were continuously incubated for 10 min at 37 °C with RPE-1 cells that were treated or not with

CBD. n = 40 cells per condition, representative of 3 independent experiments. Means ± SEM, unpaired two-sided t-test; $^{**}P < 0.002$, $^{****}P < 0.0001$. Scale bars = 10 µm. Nuclei in blue. (**F**) Effect of dominant-negative epsin 15 mutant (EPS15DN-GFP) on $\alpha_5\beta_1$ integrin endocytosis. Quantification by confocal microscopy of mAb13 (top) and 9EG7 (bottom) uptake after continuous incubation for 10 min at 37 °C with RPE-1 cells. Dashed lines indicate EPS15DN-GFP expressing cells. The GFP-negative (GFP^-) cells were used as internal controls. n = 40 cells per condition, representative of 3 independent experiments. Means ± SEM, unpaired two-sided t-test; $^{****}P<0.0001$. Scale bars = 10 µm. Nuclei in blue. (**G**) Effect of EPS15DN-GFP on Gal3 and transferrin (Tf) endocytosis. Gal3 (200 nM, top), or Tf (bottom) were continuously incubated for 10 min at 37 °C with RPE-1 cells transiently transfected with EPS15DN-GFP. Dashed lines indicate EPS15DN-GFP expressing cells. The GFP-negative (GFP^-) cells were used as internal controls. n = 40 cells per condition, representative of 3 independent experiments. Means ± SEM, unpaired two-sided t-test; $^{****}P < 0.0001$. Scale bars = 10 µm. Nuclei in blue. (**H**) $\alpha_5\beta_1$ integrin interaction with clathrin heavy chain (CHC) in RPE-1 cells. Cell surface immunoprecipitation (IP) with mAb13, 9EG7, and beads (Ø) as controls. Immunoblot (IB) for indicated antigens. n = 3 independent experiments. Means ± SEM, unpaired two-sided t-test; $^{**}P < 0.002$. (**I**) Monitoring mAb13 and 9EG7 endocytosis using lattice light sheet microscopy on AP2-GFP genome-edited RPE-1 cells. 3D projections of acquired 3D stacks of cargo (magenta) and AP2 (green). Events (pale purple) for which the indicated markers were co-tracked during endocytic uptake. Scale bars = 8 µm. (**J**) Median normalized endocytic intensity tracks of mAb13 and 9EG7 uptake from experiments as in (I). Intensities below dashed black lines were considered background. (**K**) Percentages of AP2 positive uptake events from (I) for mAb13 (727 total tracks), 9EG7 (542 total tracks), and Tf (1333 total tracks). n = 6 cells per condition. Means ± SEM, one-way ANOVA; $^{*}P < 0.05$, $^{****}P < 0.0001$. (**L**). Experiment as in (I). Left, 2D side views of cell slices perpendicular to the detection objective. Examples of mAb13 or 9EG7-positive structures that overlapped (orange squares, AP2+) or not (blue squares, AP2-) with AP2-GFP in RPE-1 cells. Scale bars = 10 µm. Right, time-resolved evolution (min:sec) of mAb13 and 9EG7 signals in relation to AP2-GFP. Examples of AP2 positive (AP2+) and AP2 negative (AP2-) tracks are shown. Images were extracted from supplementary Movies 3 and 4.

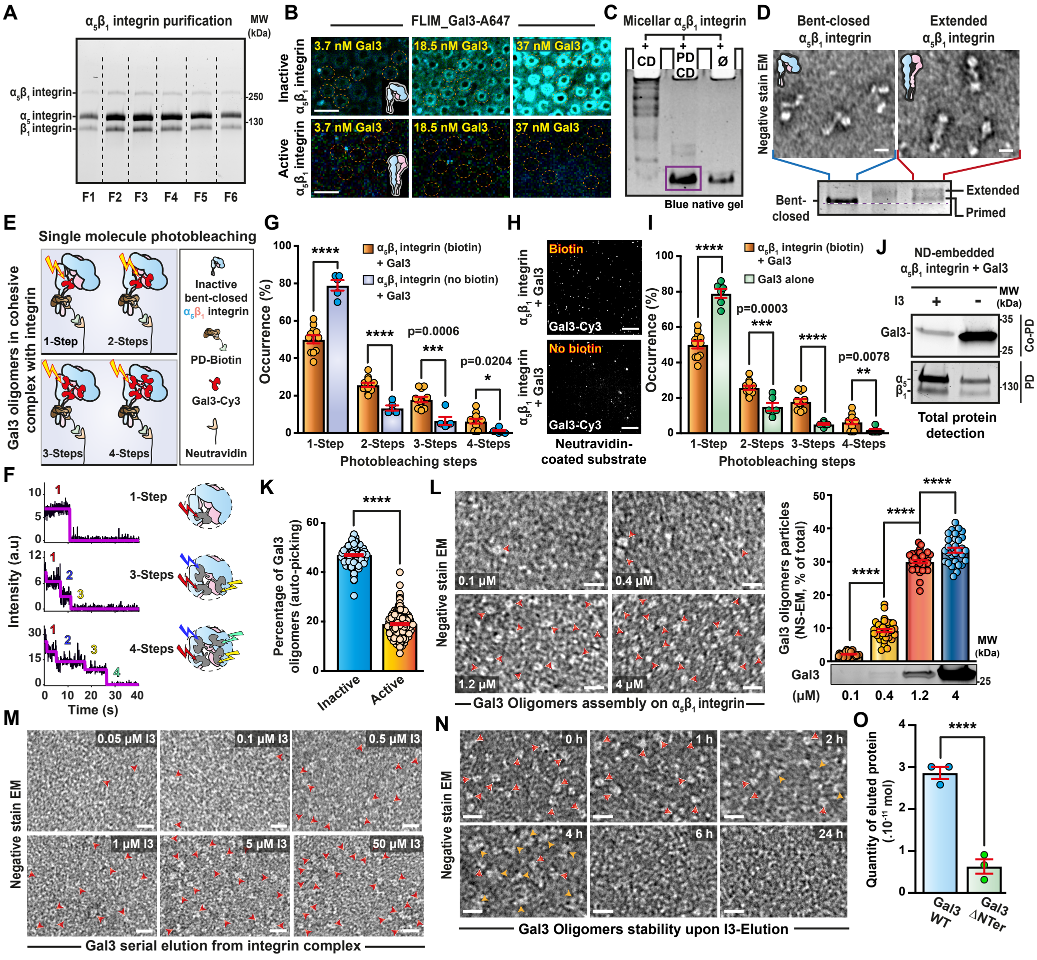

Supplementary Figure 4. Only inactive bent-closed $\alpha_5\beta_1$ integrin nucleates Gal3 oligomers. (**A**) $\alpha_5\beta_1$ integrin purification. $\alpha_5\beta_1$ integrin was solubilized from rat liver and purified in a two-step process using wheat germ agglutinin and fibronectin affinity columns. Fractions F1 to F6 from the fibronectin column were analyzed by SDS-PAGE and total protein staining to assess purity. At least 3 independent experiments with similar results were performed. (**B**) FLIM measurements on MSLB confirming the preferential interaction of Gal3 with inactive $\alpha_5\beta_1$ integrin in this minimal membrane environment. Dashed circles indicate microcavity pores. Scale bars = 5 µm. (**C**) Reconstitution of $\alpha_5\beta_1$ integrin into peptidiscs. The reconstitution from Triton X-100 micelles into peptidiscs was analyzed by blue native PAGE. Upon detergent removal using cyclodextrin (+CD) and in the absence of Apo-1 derived peptides, several bands were visible, indicative of protein aggregation. In the presence of Apo1-derived peptides and cyclodextrin (PD+CD), a major band was found (purple rectangle) that migrated at the same level as micellar integrin (Ø). These data document an efficient mono-incorporation of $\alpha_5\beta_1$ integrin into peptidiscs. (**D**) Electron micrographs of $\alpha_5\beta_1$ integrin in peptidiscs. Left: Inactive bent-closed conformation. Right: The protein was incubated with 5 mM $MnCl_2$ and 100 µM cRGD. Note the switch from the inactive bent-closed to the active extended ligand-bound conformation. Scale bars = 10 nm. At least 3 independent experiments with similar results were performed. These samples were also analyzed by blue native PAGE, which allowed to document corresponding changes in electrophoretic mobility. (**E**) Schematic of the different inactive $\alpha_5\beta_1$ integrin-Gal3 complex configurations in photobleaching experiments (PD: peptidisc). (**F**) Qualitative visualization of single Gal3 molecule photobleaching step counting in $\alpha_5\beta_1$ integrin-Gal3 complexes. (**G**) Quantification in the

indicated conditions of experiments as in (F). Peptidisc-embedded $\alpha_5\beta_1$ integrin that unspecifically bound to the neutravidin substrate (no biotin) served as control. n = 11 fields for biotin condition and n = 5 fields for no-biotin condition. Means ± SEM, unpaired two-sided t-test; *P < 0.05, ***P < 0.0002, ****P < 0.0001. (**H**) Representative images of experiments as in (E-G). Note that in the absence of biotin, fewer Gal3 spots were visible on the substrate. Scale bars = 8 µm. (**I**) Photobleaching experiments performed with Gal3-Cy3/biotin-tagged $\alpha_5\beta_1$ integrin peptidiscs, or Gal3-Cy3 alone. Bar plots show the number of steps detected via fluorescence imaging. For Gal3 alone, predominantly single-step photobleaching behavior was observed, while higher number of photo-bleaching steps were measured for $\alpha_5\beta_1$ integrin-Gal3 complexes, which we interpret as Gal3 oligomers. n = 11 fields for the $\alpha_5\beta_1$ integrin-Gal3 condition and n = 6 fields for the Gal3 alone condition. Means ± SEM, unpaired two-sided t-test; **P < 0.002, ***P < 0.0002, ****P < 0.0001. (**J**) Glycan-dependent binding of Gal3 to nanodisc (ND)-embedded inactive bent-closed $\alpha_5\beta_1$ integrin. 200 nM of untagged Gal3 were pre-incubated or not with the I3 compound, and then co-incubated with $\alpha_5\beta_1$ integrin in nanodiscs immobilized on cobalt beads. After pull-down (PD), samples were analyzed by SDS-PAGE using total protein detection mode (Stain-Free). The loss of Gal3 (co-PD) in the presence of I3 was consistent with its glycan-dependent interaction with $\alpha_5\beta_1$ integrin. (**K**) Quantification of Gal3 oligomers by particle autopicking in crYOLO [1]. $\alpha_5\beta_1$ integrin was embedded in His-tagged nanodiscs, immobilized on cobalt beads, activated with $MnCl_2$/cRGD or not, and incubated with Gal3. I3 was used to elute Gal3, which was analyzed in all conditions by negative stain EM. Oligomers were quantified by automatic particle picking. Note that erroneous scoring of irrelevant contaminating objects such as nanodiscs could not be excluded. n = 100 (for inactive $\alpha_5\beta_1$ integrin-Gal3) and n = 145 (for active $\alpha_5\beta_1$integrin-Gal3) EM-fields were analyzed. Means ± SEM, unpaired two-sided t-test; ****P < 0.0001. (**L**) Gal3 titration for oligomer assembly. Nanodisc-embedded $\alpha_5\beta_1$ integrin immobilized on beads was incubated with the indicated concentrations of Gal3. I3 was used to specifically elute Gal3. The eluate was analyzed by negative stain EM for visual quantification of oligomers, and by SDS-PAGE for total amount of Gal3 bound to $\alpha_5\beta_1$ integrin. n = 36 (for 0.1, 0.4 and 1.2 µM) and n = 31 (for 4 µM) EM-fields, with a total of 6,448 (for 0.1 µM), 6,572 (for 0.4 µM), 7,341 (for 1.2 µM) and 4,991 (for 4 µM) total particles. Means ± SEM, unpaired two-sided t-test; ****P < 0.0001. Scale bars = 20 nm for EM micrographs. (**M**) I3 titration for Gal3 elution from $\alpha_5\beta_1$ integrin. Representative negative stain EM micrographs of Gal3 oligomers visually quantified in Figure 6D. Scale bars = 20 nm. (**N**) Negative stain EM micrographs of Gal3 oligomers as in (L) (Gal3 4 µM), that were either analyzed directly (0 h), or kept in solution at 4 °C for analysis at the indicated time points. Note that Gal3 oligomers (red arrowheads) were stable up to 2 h after elution, then started to condense (2-4 h after elution, orange arrowheads) to finally disassemble into monomers after 6-24 h. Scale bars = 20 nm. (**O**) Quantification of Gal3 and Gal3ΔNter binding to inactive bent-closed $\alpha_5\beta_1$ integrin in nanodiscs. After binding (4 µM) and elution, eluates were analyzed by denaturing SDS-PAGE, and total protein signals (stain-free detection) were quantified. n = 3 independent experiments. Means ± SEM, unpaired two-sided t-test; ****P < 0.0001.

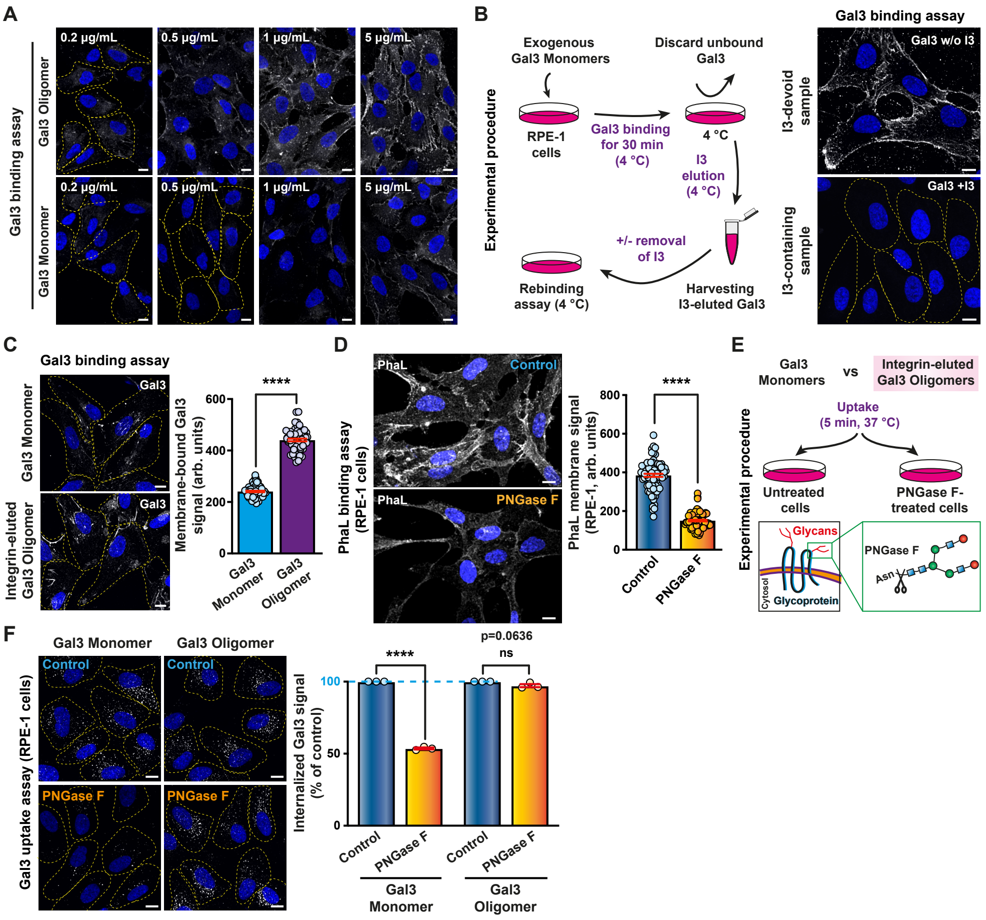

Supplementary Figure 5. Assembled Gal3 oligomers acquire new binding avidity properties. (**A**) Binding of Gal3 monomers versus cell-eluted Gal3 oligomers onto RPE-1 cells. Confocal microscopy images, quantification in Figure 7E. Scale bars = 10 µm. Nuclei in blue. (**B**) Glycan-dependent rebinding of cell-eluted Gal3 oligomers. After I3 elution from RPE-1 cells, Gal3 oligomers were either desalted to remove I3 before rebinding, or directly incubated with RPE-1 cells. Left: Schematic of the experimental procedure. Right: Representative images. Note that the presence of I3 completely abolished re-binding of preformed Gal3 oligomers. In at least 3 independent experiments, similar results were observed. Scale bars = 10 µm. Nuclei in blue. (**C**) Re-binding onto cells of Gal3 oligomers eluted from $\alpha_5\beta_1$ integrin. RPE-1 cells were incubated for 30 min at 4 °C with the same mass equivalents of Gal3 oligomers eluted from pure $\alpha_5\beta_1$ integrin, or of Gal3 monomers. Note that Gal3 oligomers that were pre-formed on $\alpha_5\beta_1$ integrin bound more efficiently than Gal3 monomers, as observed in Figure 7D for Gal3 oligomers that were pre-formed on cells. Increased oligomer binding is therefore not due to contaminating cellular components. n = 68 (for Gal3 monomer) and n = 63 (for Gal3 oligomer) cells, representative of 3 independent experiments. Means ± SEM, unpaired two-sided t-test; ****$P < 0.0001$. Scale bars = 10 µm. Nuclei in blue. (**D**) Effectiveness of PNGase F treatment. The PhaL lectin interacts with branched N-glycan. The substantial inhibition of its binding to PNGase F treated RPE-1 cells documented an efficient removal of cell surface N-glycans. n = 89 cells were analyzed, representative of 3 independent experiments. Means ± SEM, unpaired two-sided t-test; ****$P < 0.0001$. Scale bars = 10 µm. Nuclei in blue. (**E**) Schematic for the use of PNGase F to assess the role of N-glycans in monomeric versus

oligomeric Gal3 uptake into cells. (**F**) Gal3 monomer versus $\alpha_5\beta_1$ integrin-eluted Gal3 oligomer uptake into RPE-1 cells that have been treated or not with PNGase F, as described in (E). Note that at the same mass equivalents, integrin-eluted Gal3 oligomer uptake into PNGase F treated cells was much more efficient than that of monomeric Gal3, as observed for cell-eluted Gal3 oligomers in Figure 7D. n = 3 independent experiments. Means ± SEM, unpaired two-sided t-test; ns = $P > 0.05$, ****$P < 0.0001$. Scale bars = 10 µm. Nuclei in blue.

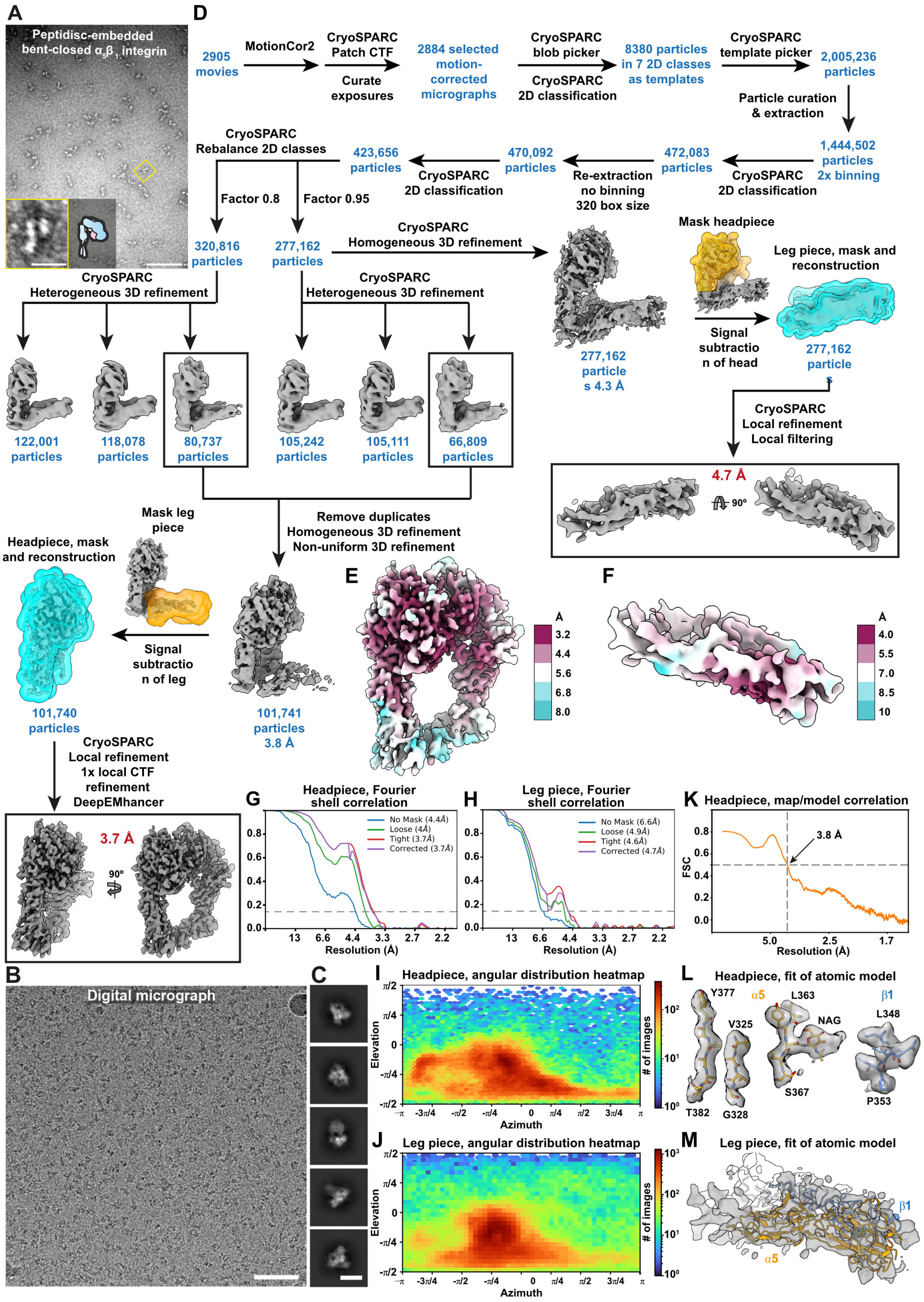

Supplementary Figure 6. Cryo-EM processing of peptidisc-embedded $\alpha_5\beta_1$ integrin. (**A**) Negative stain EM micrograph showing efficient mono-insertion of $\alpha_5\beta_1$ integrin into peptidiscs. Scale bar = 100 nm and 20 nm for zoom-in. (**B**) Typical digital micrograph at 2.0 µm defocus, recorded with a Falcon-3 camera at 300 kV. Scale bar = 50 nm. (**C**) Representative 2D class averages of $\alpha_5\beta_1$ integrin, showing different projection views. Scale bar = 10 nm. (**D**) Schematic of the cryo-EM data processing pipeline, including signal subtraction and local refinements of the headpiece and leg piece. (**E,F**) Density maps of the headpiece (E) and leg piece (F) of $\alpha_5\beta_1$ integrin, colored by local resolution as calculated in CryoSPARC. (**G,H**) Fourier shell correlation (FSC) curves of the final local refinements of cryo-EM densities of headpiece (G) and leg piece (H). (**I,J**) Angular distribution heatmaps of the particles used in the final refinements of headpiece (I) and leg piece (J). (**K**) Map-to-model correlation of the

headpiece of $\alpha_5\beta_1$ integrin (α_5, residues 94 - 691, β_1, 25-504). (**L**) Fit of atomic model in selected parts of the headpiece (left: α_5, β-propeller, middle: α_5, glycan at N365, right: β_1, α-helix). (**M**) Rigid body fit of homology model of leg piece of rat $\alpha_5\beta_1$ integrin (α_5, residues 692-1041; β_1, residues 505-720) in the cryo-EM density of the leg piece.

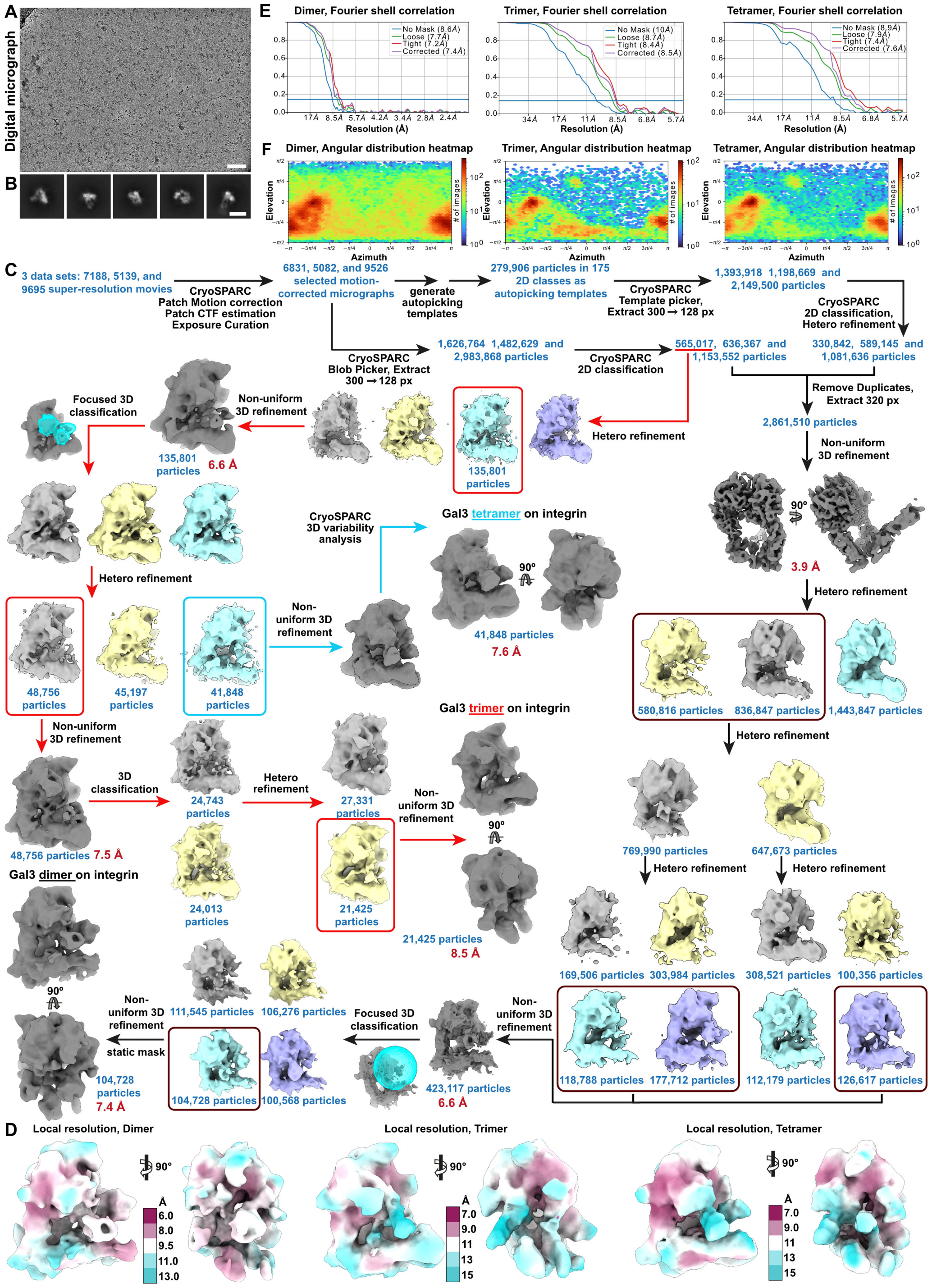

Supplementary Figure 7. Cryo-EM processing of nanodisc-embedded $\alpha_5\beta_1$ integrin-Gal3 complexes. (**A**) A subset of 260 typical digital micrographs at 1.9 µm defocus, recorded with a Gatan K3 camera at 300 kV. Scale bar = 50 nm. (**B**) Representative 2D class averages of the $\alpha_5\beta_1$ integrin-Gal3 complex, showing different projection views. Scale bar = 10 nm. (**C**) Schematic of the cryo-EM data processing pipeline of the nanodisc-embedded $\alpha_5\beta_1$ integrin-Gal3 complex. The processing pathways of $\alpha_5\beta_1$ integrin with Gal3 dimer, trimer and tetramer are illustrated in black, red and cyan, respectively. (**D**) Cryo-EM density maps of the $\alpha_5\beta_1$ integrin-Gal3 complexes for a bound Gal3 dimer, trimer, and tetramer, respectively, colored by local resolution as obtained in CryoSPARC. (**E,F**) Fourier shell correlation (FSC) curves (E)

and angular distribution heatmaps (F) for the final non-uniform refinements of $\alpha_5\beta_1$ integrin-Gal3 complexes for a bound Gal3 dimer, trimer and tetramer.

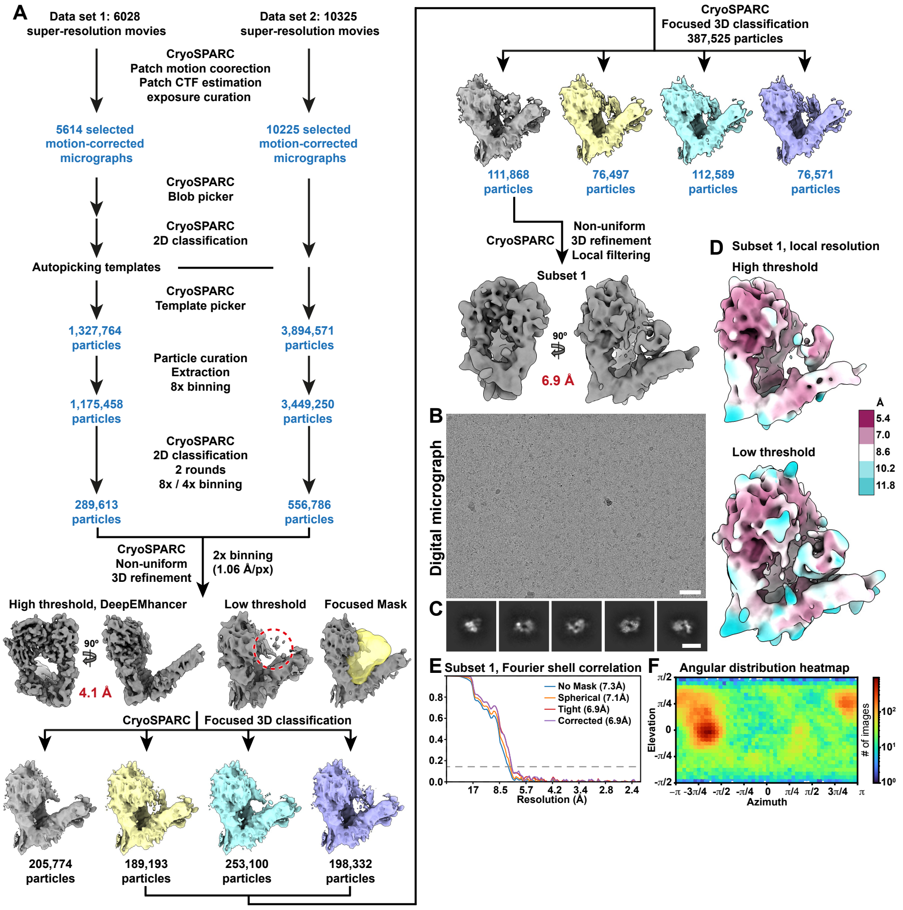

Supplementary Figure 8. Cryo-EM processing of peptidisc-embedded $\alpha_5\beta_1$ integrin-Gal3 complexes. (**A**) Schematic of the cryo-EM data processing pipeline of $\alpha_5\beta_1$ integrin-Gal3 complexes. With 111,868 particles, subset 1 with the most clearly defined density between head and leg pieces accounted for 13% of the total data set, indicating that only a fraction of $\alpha_5\beta_1$ integrin in this preparation was competent for stable Gal3 binding and oligomerization. (**B**) Typical digital micrograph at 2.0 µm defocus, recorded with a Gatan K3 camera at 300 kV. Scale bar = 50 nm. (**C**) Representative 2D class averages of $\alpha_5\beta_1$ integrin-Gal3 complexes, showing different projection views. Scale bar = 10 nm. (**D**) Cryo-EM density map of subset 1 of $\alpha_5\beta_1$ integrin-Gal3 complexes, colored by local resolution as obtained in CryoSPARC. (**E,F**) Fourier shell correlation (FSC) curves (E) and angular distribution heatmap (F) of the final non-uniform refinement of subset 1 of $\alpha_5\beta_1$ integrin-Gal3 complexes.

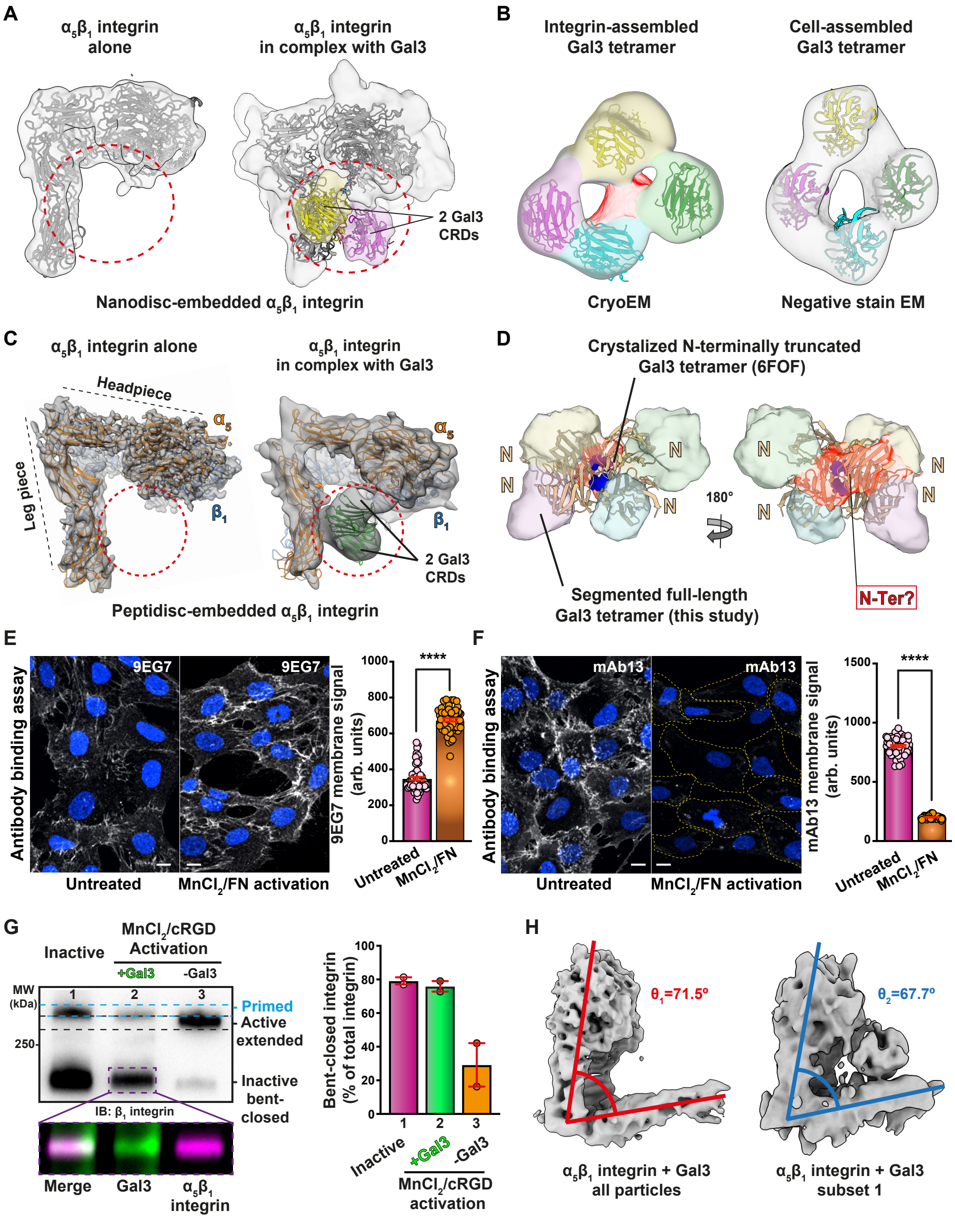

Supplementary Figure 9. Gal3 clamps the inactive bent-closed conformational state of $\alpha_5\beta_1$ integrin. (**A**) Cryo-EM density maps of nanodisc-embedded a_5b_1 integrin (grey) of the complex data set in Supplementary Figure 7, either without Gal3 (left, from a subset of 10,498 particles), or in complex with Gal3 (right, subset corresponding to Gal3 dimer with 104,728 particles)., either without Gal3 (left), or in complex with Gal3 (right, subset corresponding to Gal3 dimer with 104,728 particles). The red dashed circle indicates a region with additional cryo-EM densities in which two Gal3 CRDs (yellow and purple) were fitted. (**B**) Comparison of isolated Gal3 tetramers that were nucleated on purified $\alpha_5\beta_1$ integrin (left, cryo-EM) or on cells (right, negative stain EM). Both overall resemble each other in size and shape. Densities that could correspond to Gal3's N-termini and that connect the four CRDs are resolved only in the cryo-EM data (left, translucent red). (**C**) Cryo-EM density map of peptidisc-embedded $\alpha_5\beta_1$ integrin (orange for α_5 and blue for β_1 subunits) alone (left), or in complex with Gal3 (right). The red dashed circle indicates a region where additional cryo-EM densities were only observed in the presence of Gal3 (CRD in green). (**D**) Fit of a N-terminal deletion mutant Gal3 tetramer crystallized under non-native conditions (PDB 6FOF) into the density map of full-length Gal3 tetramers

segmented from the $\alpha_5\beta_1$ integrin-Gal3 complex in Figure 8C. Note the poor overlap between both structures. (**E,F**) $\alpha_5\beta_1$ integrin activation on cells. RPE-1 cells were incubated at 4 °C with or without 1 mM $MnCl_2$ and 5 µg/mL soluble fibronectin (FN), then with 9EG7 (E) or mAb13 (F) antibodies, fixed and immunolabeled. Quantification showed an increased 9EG7 labeling in the $MnCl_2$/FN condition (E), and a concomitantly decreased mAb13 labeling (F). n = 60 cells per condition, representative of 3 independent experiments. Means ± SEM, unpaired two-sided t-test; ****$P < 0.0001$. Scale bars = 10 µm. Nuclei in blue. (**G**) Effect of Gal3 on $\alpha_5\beta_1$ integrin activation. Micellar inactive bent-closed $\alpha_5\beta_1$ integrin was pre-incubated or not with 2 µM of Gal3-Alexa488 and then incubated with 5 mM $MnCl_2$ and 100 µM cRGD. Samples were run on semi-native gels, and bands were detected by fluorescence for Gal3, or anti-β_1 integrin immunoblotting. Non-activated $\alpha_5\beta_1$ integrin showed a dominant lower band, likely the bent-closed conformer (lane 1), and a weaker upper band, likely representing the primed conformational state (delimited by blue dashed line). Upon incubation with $MnCl_2$ and cRGD, a clear band switch was observed in favor of a new upper band (delimited by black dashed lines), likely corresponding to the active extended ligand-bound conformation (lane 3). Upon pre-incubation with Gal3, this band switch was strongly reduced (lane 2). Note that Gal3 and $\alpha_5\beta_1$ integrin overlapped in the lower band of lane 2. In all conditions, the lower bands (i.e., the inactive bent-closed conformer) were then quantified from 2 independent experiments as the percentage of total $\alpha_5\beta_1$ integrin. Means ± SEM are shown. (**H**) Comparison of the angles between the head and leg pieces of $\alpha_5\beta_1$ integrin in the presence or absence of Gal3. Unsharpened maps are shown. Left: Density map of the full data set, including many $\alpha_5\beta_1$ integrin heterodimers without bound Gal3, and non-cohesive $\alpha_5\beta_1$ integrin-Gal3 complexes. Right: Subset 1 with visible additional densities that fitted with Gal3 binding to $\alpha_5\beta_1$ integrin. A larger angle was observed in the full data set ($\theta_1 = 71.5°$ all particle) compared to subset 1 ($\theta_2 = 67.7°$, $\Delta\theta = 3.8°$), indicating that Gal3 binding led to a compaction of $\alpha_5\beta_1$ integrin, likely by clamping the protein in the inactive bent-closed conformational state, that prevents activation.

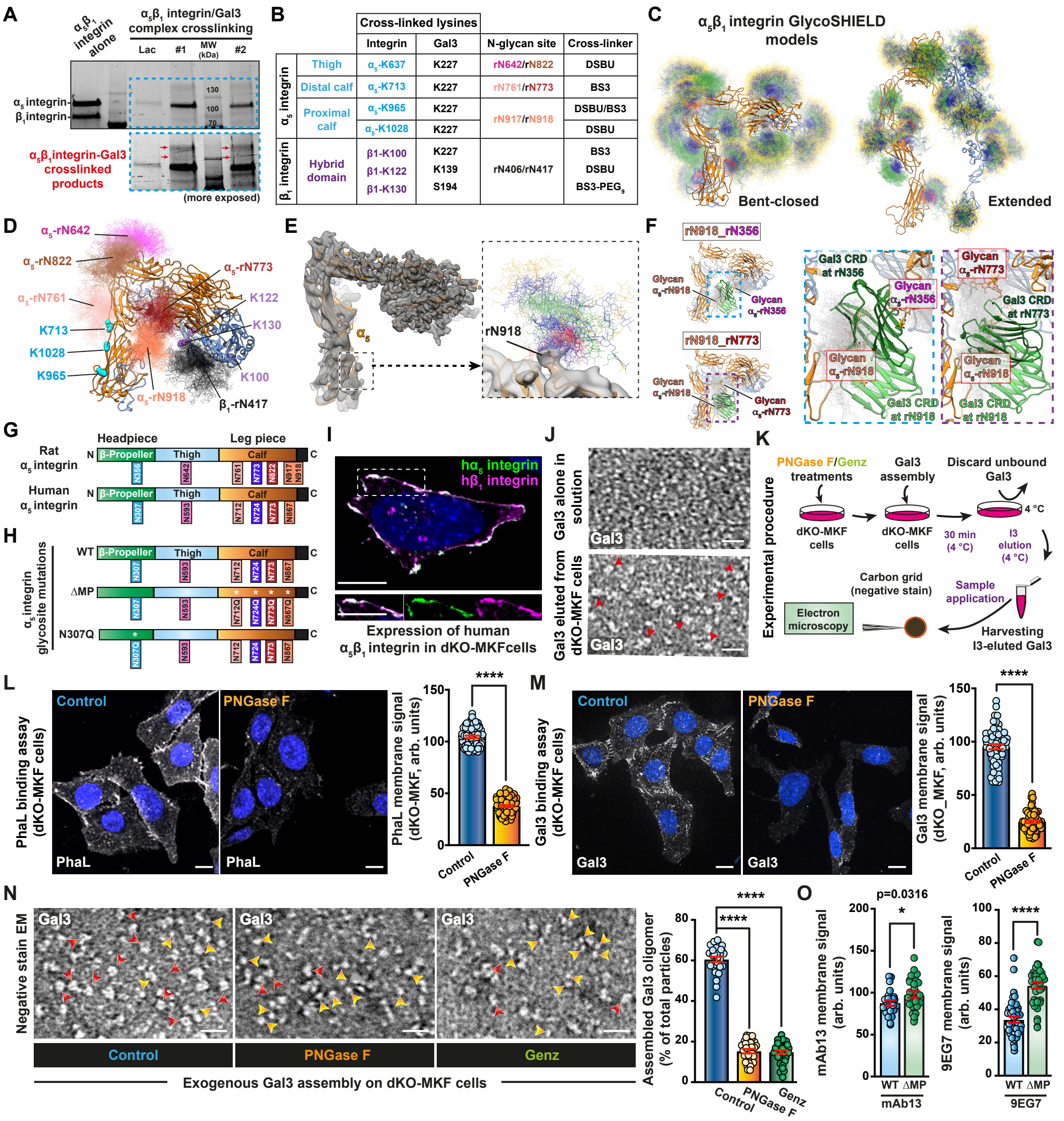

Supplementary Figure 10. Glycans for the functional recognition of bent-closed $\alpha_5\beta_1$ integrin by oligomerization competent Gal3. We use "r" or "h" tags in front of each N-glycosylations site to indicate its position in $\alpha_5\beta_1$ integrin of rat or human origins, respectively. (**A**) Cross-linking data analysis by SDS-PAGE. 2 cross-linking reactions (lanes #1 and #2) between $\alpha_5\beta_1$ integrin and Gal3 were analyzed. The zoomed box (blue dashed line) with stronger exposure revealed bands with lower electrophoretic mobility (red arrows) than α_5 and β_1 integrin chains, likely representing cross-linking products. These bands were lost in the presence of lactose (Lac), which indicated that their appearance relied on glycan-Gal3 interaction. (**B**) Cross-linking mass spectrometry analysis on the $\alpha_5\beta_1$ integrin-Gal3 complex. Cross-linked lysine positions from trans-peptides (Gal3 and $\alpha_5\beta_1$ integrin) as well as the cross-linkers used are reported in the table. N-glycans that are spatially positioned in a way such as to be compatible with cross-linked lysines and cross-linker length are also indicated. (**C**) 3D-structure of $\alpha_5\beta_1$ integrin with GlycoSHIELD models of all identified N-glycans in rat $\alpha_5\beta_1$ integrin, in either the bent-closed (left) or the extended (right) conformations. Blue, N-acetylglucosamine; green, mannose; red, fucose; yellow, galactose. 100 possible conformations are shown for each glycan position. (**D**) Cross-linked lysines (in blue for α_5 integrin and purple for β_1 integrin) and complex glycans in their vicinity (in red tones for α_5 integrin and black for β_1 integrin) were projected onto the $\alpha_5\beta_1$ integrin 3D-model structure. GlycoSHIELD

conformations were computed for each of these glycans, and 100 possible conformations are shown each. (**E**) $\alpha_5\beta_1$ integrin model in cryo-EM density map. The box highlights the proximal leg piece of α_5 integrin with a density that can be ascribed to a glycan at position α_5-rN918. Glycan conformations on α_5-rN918 (42 projections by GlycoSHIELD) are shown. (**F**) Model of peptidisc-embedded $\alpha_5\beta_1$ integrin-Gal3 complex. GlycoSHIELD conformations are shown for glycans at α_5-rN356 and α_5-rN918 (Top left and blue dashed lined zoomed view), or at α_5-rN773 and α_5-rN918 (Bottom left and purple dashed line zoomed view). Two Gal3 CRDs modeled within the cryo-EM density are highlighted along with glycans that fit best. The other GlycoSHIELD conformations are shown in light gray. (**G**) Linear alignment of key N-glycosylation sites in rat and human α_5 integrins, as identified by cross-linking proteomics and cryo-EM. (**H**) Linear representation of human α_5 integrin, and N-glycosylation sites whose role in Gal3 binding are suggested by cross-linking proteomics and cryo-EM. N to Q mutations in ΔMP and α_5-hN307Q are indicated by white stars. (**I**) Transient expression of human α_5 integrin-GFP (green) and human β_1 integrin-Halo tag (red) in dKO-MKF cells. Note that both chains co-localize at the plasma membrane, indicating that these have heterodimerized. At least 3 independent experiments with similar results were performed. Scale bars = 10 µm. Nuclei in blue. (**J**) Gal3 oligomers assembly on dKO-MKF cells. After binding of monomeric Gal3 on dKO-MKF cells for 30 min at 4 °C, Gal3 was eluted with I3 and spotted on EM grids for negative stain EM analysis. Monomeric Gal3 in solution was also loaded as control. Red arrowheads indicate Gal3 oligomers. At least 3 independent experiments were performed, and all gave similar results. Scale bars = 20 nm. (**K**) Schematic of experiments for Gal3 oligomers assembly on dKO-MKF upon PNGase F/Genz treatments. (**L**, **M**) Efficiency of PNGase F treatment on dKO-MKF cells. Both branched N-glycans binder PhaL (L) and monomeric Gal3 (M) showed substantial binding inhibition upon PNGase F treatment, indicative of an efficient removal of N-glycans from the cell surface. n = 60 cells for each condition, representative of 3 independent experiments. Means ± SEM, unpaired two-sided t-test; ****$P < 0.0001$. Scale bars = 10 µm. (**N**) Gal3 samples that were I3-eluted from dKO-MKF in different conditions (as described in K) were analyzed by negative staining EM, and Gal3 oligomers were quantified as percentage of total particles by visual picking. Control: n = 28 EM-fields with 8,832 total particles; PNGase F: n = 28 EM-fields with 8,510 total particles; Genz: n = 33 EM-fields with 6,357 total particles. As for Gal3 eluted from RPE-1 cells (Figure 7I), removal of N-glycans (PNGaseF) or inhibition of GSL expression (Genz) clearly affected the formation of Gal3 oligomers. Red and yellow arrowheads indicate oligomers and monomers of Gal3, respectively. 3 independent experiments. Means ± SEM, one-way ANOVA; ****$P < 0.0001$. Scale bars = 20 nm. (**O**) Expression of $\alpha_5\beta_1$ integrin at the cell surface. mAb13 or 9EG7 antibody binding experiments were performed at 4 °C in wildtype $\alpha_5\beta_1$ integrin or ΔMP α_5 integrin/wildtype β_1 integrin-expressing dKO-MKF cells. Plasma membrane signals of mAb13 and 9EG7 were quantified. n = 30 (for mAb13) and n = 40 (for 9EG7) cells were analyzed, representative of 3 independent experiments. Means ± SEM, unpaired two-sided t-test; *$P < 0.05$, ****$P < 0.0001$.

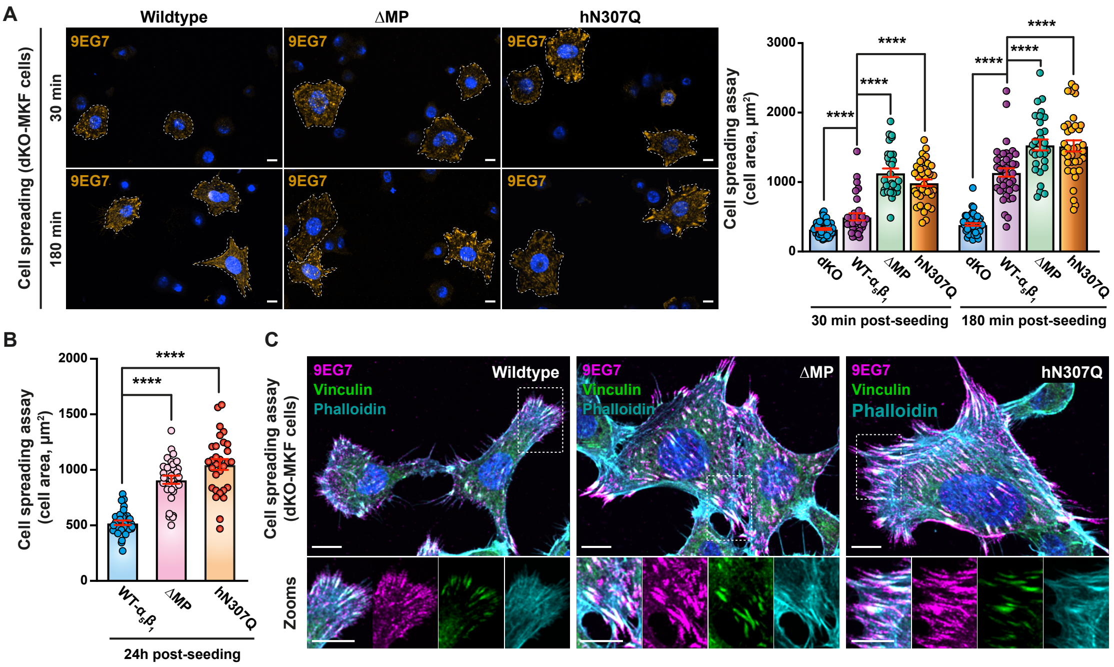

Supplementary Figure 11. Cell spreading relies on the functional expression of N-glycans. (**A**) Effect of mutating $\alpha_5\beta_1$ integrin N-glycosylation sites on adhesion and spreading of dKO-MKF cells. dKO-MKF cells expressing wildtype $\alpha_5\beta_1$ integrin, ΔMP α_5 integrin/wildtype β_1 integrin, or hN307Q α_5 integrin/wildtype β_1 integrin, were harvested 24 h after transfection, and seeded for 30 min or 180 min at 37 °C on fibronectin-coated glass coverslips. Cells were then incubated with 9EG7 antibody for 30 min at 4 °C. Plasma membrane signal of 9EG7 was used to define cell area, which was measured and quantified by confocal microscopy. 30 min: n = 50 (for untransfected dKO), n = 31 (for WT and ΔMP) and n = 39 (for hN307Q) cells; 180 min: n = 52 (for dKO, untransfected cells), n = 40 (for WT), n = 30 (for ΔMP) and n = 35 (for hN307Q) cells per condition. Representative of 3 independent experiments. Means ± SEM, one-way ANOVA; ****P < 0.0001. Scale bars = 10 µm. Nuclei in blue. (**B**) Experiments as in (A), assessing cell adhesion and spreading after 24 h on fibronectin-coated glass coverslips. n = 30 cells per condition. Representative of 3 independent experiments. Means ± SEM, one-way ANOVA; ****P < 0.0001. Scale bars = 10 µm. Nuclei in blue. (**C**) Experiment as in (A) performed 6 h after seeding, with immunolabeling for the focal adhesion protein vinculin, and with phalloidin-labeling for F-actin. At least 3 independent experiments were performed, which all gave similar results. Scale bars = 10 µm. Nuclei in blue.

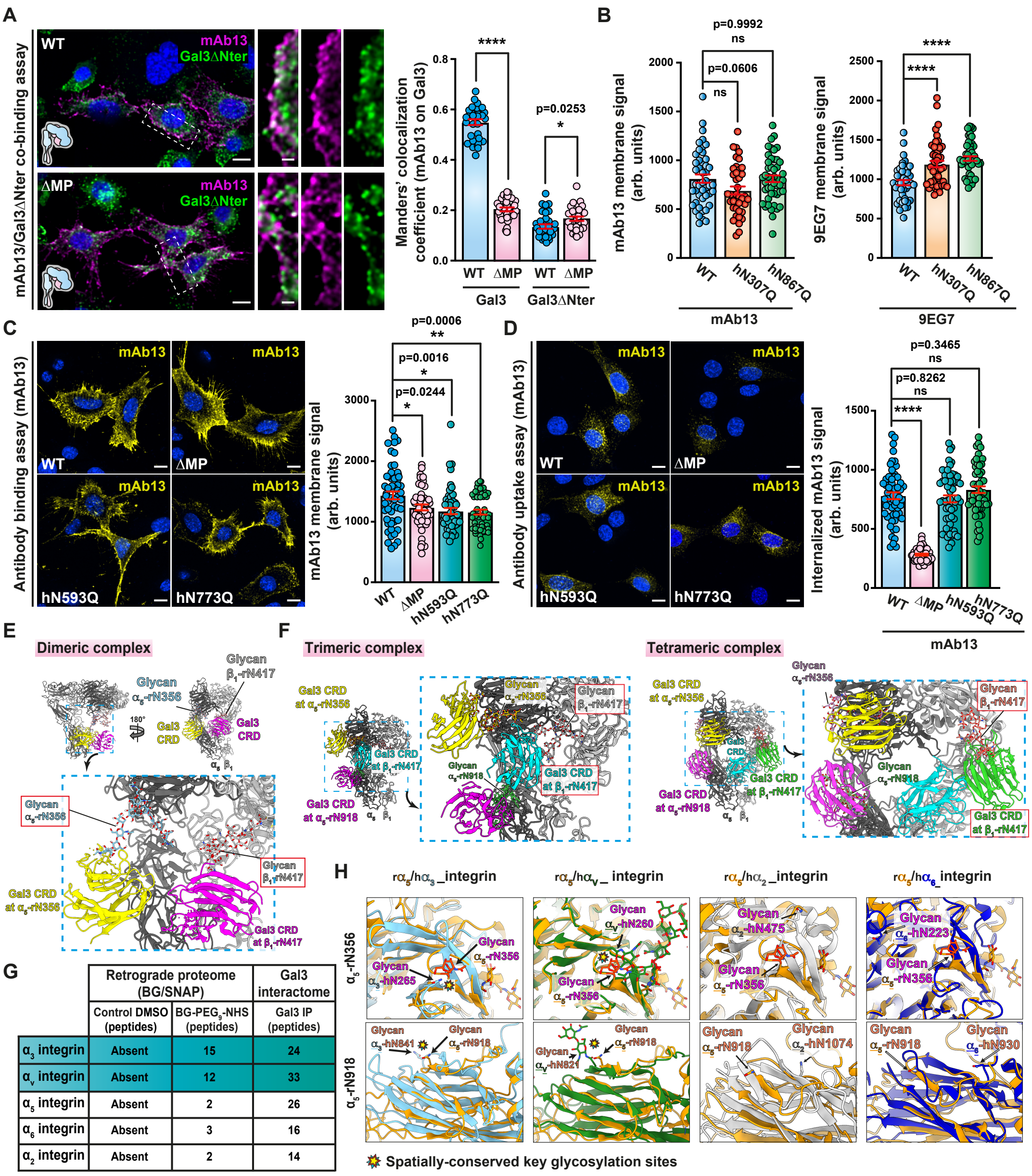

	Retrograde proteome (BG/SNAP)		Gal3 interactome
	Control DMSO (peptides)	BG-PEG$_3$-NHS (peptides)	Gal3 IP (peptides)
α_3 integrin	Absent	15	24
α_v integrin	Absent	12	33
α_5 integrin	Absent	2	26
α_6 integrin	Absent	3	16
α_2 integrin	Absent	2	14

Supplementary Figure 12. Towards a glycan signature for GL-Lect driven endocytosis. (**A**) Gal3 or Gal3ΔNter (200 nM) and mAb13 or 9EG7 co-binding experiments on dKO-MKF cells in wildtype $\alpha_5\beta_1$ integrin versus ΔMP α_5 integrin/wildtype β_1 integrin conditions. Colocalization of mAb13 and 9EG7 with Gal3 or Gal3ΔNter was quantified. n = 33 cells per condition, representative of 3 independent experiments. Means ± SEM, unpaired two-sided t-test; *P < 0.02, ****P < 0.0001. Scale bars = 5 µm. Nuclei in blue. (**B**) Expression of $\alpha_5\beta_1$ integrin at the cell surface. mAb13 or 9EG7 binding experiments were performed at 4 °C on dKO-MKF cells in the wildtype $\alpha_5\beta_1$ integrin versus α_5-hN307Q or α_5-hN867Q integrin/wildtype β_1 integrin conditions. Plasma membrane signals of mAb13 and 9EG7 were quantified. n = 46 (for WT-mAb13), n = 40 (for hN307Q-mAb13), n = 48 (for hN867Q-mAb13), n = 45 (for WT-9EG7), n = 48 (for hN307Q-9EG7) and n = 50 (for hN867Q-9EG7) cells, representative of 3 independent experiments. Means ± SEM, one-way ANOVA; ns = P > 0.5, ****P < 0.0001. (**C**) mAb13 binding to dKO-MKF cells as in (B), for ΔMP, hN593Q and hN773Q α_5 integrin/wildtype β_1 integrin. n = 57 (for WT), n= 47 (for ΔMP), n = 51 (for hN593Q), and n = 52 (for hN773Q) cells, representative of 3 independent experiments. Means

± SEM, one-way ANOVA; *P < 0.05, **P < 0.0021. Scale bars = 10 μm. Nuclei in blue. (**D)** Endocytosis with α_5-ΔMP, α_5-hN593Q, and α_5-hN773Q integrin/wildtype β_1 integrin. Continuous incubation for 10 min at 37 °C of mAb13 with dKO-MKF cells under the indicated conditions. Non-internalized antibodies were removed by acid wash. n = 60 (for WT), n = 45 (for ΔMP), n = 57 (for hN593Q) and n = 56 (for hN773Q) cells, representative of 3 independent experiments. Means ± SEM, one-way ANOVA; ns = P > 0.5, ****P < 0.0001. Scale bars = 10 μm. Nuclei in blue. (**E**) Fitted model of nanodisc-embedded $\alpha_5\beta_1$ integrin in complex with Gal3 (CRD, PDB 1KJL) dimer, modeled to glycans at positions α_5-rN356 (hN307) and β_1-rN417. GlycoSHIELD conformations that fit best with Gal3 CRDs are highlighted (dashed blue line insets). (**F**) Fitted model of nanodisc-embedded $\alpha_5\beta_1$ integrin in complex with Gal3 (CRD, PDB 1KJL) trimers and tetramers, respectively. β_1-rN417 is consistently found to be compatible with one Gal3 CRD. (**G**) Table summarizing the presence of α integrins in retrograde proteome (BG-PEG$_9$-NHS) and Gal3 interactome (Gal3 IP)[2]. DMSO treated cells served as control. Note that α3 and αv integrins were highly abundant in both mass spectrometry lists. (**H**) Overlay of 3D integrin structures at key N-glycosylation sites identified for rat α_5 integrin (orange) with human α chains from other integrins. The 3D-model of rat α_5 integrin (current study) was overlayed with AlphaFold2 models of human α_V and α_3 integrins, which were highly abundant in retrograde proteome and Gal3 interactome. For both, predicted N-glycosylation sites were found to be conserved and spatially near to key α_5-rN356 (α_V-hN260, α_3-hN265) and α_5-rN918 (α_V-hN821, α_3-hN841) sites. For α_2 and α_6 integrins that were less abundant in retrograde proteome and Gal3 interactome, predicted N-glycosylation sites were found to be less/not conserved and spatially distant/not present to key α_5-N356 (α_2-hN475, α_6-hN223) and α_5-rN918 (α_2-hN1074, α_6-hN930) sites.

Supplementary Table 1: Cryo-EM data collection, refinement and validation statistics.

	Neuraminidase-treated rat α5β1 integrin	Neuraminidase-treated, peptidisc-embedded rat α5β1 integrin - Gal3 complex	Neuraminidase-treated, nanodisc-embedded rat α5β1 integrin - Gal3 complex
**Data collection and processing**			
Magnification	96,000	81,000	81,000
Voltage (kV)	300	300	300
Camera	TFS Falcon 3	Gatan K3	Gatan K3
Electron exposure (e–/Å^2)	42	61	80.3
Defocus range (µm)	-0.8 – -2.0	-1.0 – -2.8	-1.3 – -2.6
Pixel size (Å)	0.832	1.06 (0.53 super resolution)	1.06 (0.53 super resolution)
Micrographs used	2884	15,839	21,439
Total extracted particle images	1,444,502	4,624,708	6,093,261
Refined particle images	470,092	846,399	2,861,510
Final particle images	101,740 (head) 277,162 (leg)	111,868	104,728 / 21,425 / 41,848 (dimer / trimer / tetramer)
Map resolution	3.7 (head); 4.7 (leg)	6.9	7.4 / 8.5 / 7.5 (dimer / trimer / tetramer)
FSC threshold	0.143	0.143	0.143
Map resolution range (Å)	3.2 – 8.0 (head) 4.0 - 10.0 (leg)	5.4 – 11.8	6.0 – 13.0 (dimer) 7.0 – 15.0 (trimer) 7.0 – 15.0 (tetramer)
**Model Refinement**	**head:** α5, 94-691, β1, 25-504		
Refinement package	Phenix dev 4778		
Model resolution (Å)	3.8		
FSC threshold	0.5		
Map sharpening *B* factor (Å^2)	-144.0		
Model composition			
Non-hydrogen atoms	8324		
Protein residues	1078		
Ligands	8		
B factors (Å^2)			
Protein	85.55		
Ligand	78.53		
R.m.s. deviations			
Bond lengths (Å)	0.004		
Bond angles (°)	0.799		
Validation			
MolProbity score	2.18		
Clashscore	15.56		
EMRinger score	1.92		
Poor rotamers (%)	1.12		
Ramachandran plot			
Favored (%)	93.02		
Allowed (%)	6.98		
Disallowed (%)	0.00		

REFERENCES

1. Wagner, T., Merino, F., Stabrin, M., Moriya, T., Antoni, C., Apelbaum, A., Hagel, P., Sitsel, O., Raisch, T., Prumbaum, D., et al. (2019). SPHIRE-crYOLO is a fast and accurate fully automated particle picker for cryo-EM. Commun. Biol. *2*, 218. 10.1038/s42003-019-0437-z.
2. Lakshminarayan, R., Wunder, C., Becken, U., Howes, M.T., Benzing, C., Arumugam, S., Sales, S., Ariotti, N., Chambon, V., Lamaze, C., et al. (2014). Galectin-3 drives glycosphingolipid-dependent biogenesis of clathrin-independent carriers. Nat. Cell Biol. *16*, 595-606. 10.1038/ncb2970.
