# Supplementary material for: Spatial N-glycan rearrangement on α5β1 integrin nucleates galectin-3 oligomers to determine endocytic fate
Source: Nat Commun. 2025 Oct 27;16:9461. doi: 10.1038/s41467-025-64523-7 (PMC12559291; doi:10.1038/s41467-025-64523-7)
Supplement: Supplementary file 2 — Description of Additional Supplementary File [file 41467_2025_64523_MOESM2_ESM.pdf]

Description of Additional Supplementary Files

Supplementary data 1 : a5b1 integrin-Gal3 complex cross-linking mass spectrometry, set-up description and data sets

Supplementary data 2 : PDB validation report for a5b1 integrin-Gal3 complex (Gal3-Dimer)
Supplementary data 3 : PDB validation report for a5b1 integrin-Gal3 complex (Gal3-Trimer)
Supplementary data 4 : PDB validation report for a5b1 integrin-Gal3 complex (Gal3-Tetramer)

Supplementary movie 1 : 3D movie of the dynamic co-tracking of mAb13/Gal3 monitored by lattice light sheet microscopy, related to Figure 2D.

Supplementary movie 2 : 3D movie of the dynamic co-tracking of 9EG7/Gal3 monitored by lattice light sheet microscopy, related to Figure 2D.

Supplementary movie 3 : 3D movie of mAb13 antibody endocytosis as monitored by lattice light sheet microscopy, related to Supplementary Figure 3I, top.

Supplementary movie 4 : 3D movie of 9EG7 antibody endocytosis as monitored by lattice light sheet microscopy, related to Supplementary Figure 3I, bottom.

Supplementary movie 5 : 2D movie of time-resolved mAb13/AP2-positive and mAb13/AP2-negative endocytosis imaged by lattice light sheet microscopy, related to Supplementary Figure 3L, top.

Supplementary movie 6 : 2D movie of time-resolved 9EG7/AP2-positive and 9EG7/AP2-negative endocytosis imaged by lattice light sheet microscopy, related to Supplementary Figure 3L, bottom.
