# Supplementary material for: Spatial N-glycan rearrangement on α5β1 integrin nucleates galectin-3 oligomers to determine endocytic fate
Source: Nat Commun. 2025 Oct 27;16:9461. doi: 10.1038/s41467-025-64523-7 (PMC12559291; doi:10.1038/s41467-025-64523-7)
Supplement: Supplementary file 13 — Reporting summary [file 41467_2025_64523_MOESM13_ESM.pdf]

nature portfolio

Corresponding author(s): Ludger Johannes; Massiullah Shafaq-Zadah; Daniel Roderer

Last updated by author(s): 05/09/2025

Reporting Summary

Nature Portfolio wishes to improve the reproducibility of the work that we publish. This form provides structure for consistency and transparency in reporting. For further information on Nature Portfolio policies, see our Editorial Policies and the Editorial Policy Checklist.

Statistics

For all statistical analyses, confirm that the following items are present in the figure legend, table legend, main text, or Methods section.

n/a	Confirmed	
☐	☒	The exact sample size (*n*) for each experimental group/condition, given as a discrete number and unit of measurement
☐	☒	A statement on whether measurements were taken from distinct samples or whether the same sample was measured repeatedly
☐	☒	The statistical test(s) used AND whether they are one- or two-sided<br>*Only common tests should be described solely by name; describe more complex techniques in the Methods section.*
☒	☐	A description of all covariates tested
☒	☐	A description of any assumptions or corrections, such as tests of normality and adjustment for multiple comparisons
☐	☒	A full description of the statistical parameters including central tendency (e.g. means) or other basic estimates (e.g. regression coefficient) AND variation (e.g. standard deviation) or associated estimates of uncertainty (e.g. confidence intervals)
☐	☒	For null hypothesis testing, the test statistic (e.g. *F*, *t*, *r*) with confidence intervals, effect sizes, degrees of freedom and *P* value noted<br>*Give P values as exact values whenever suitable.*
☒	☐	For Bayesian analysis, information on the choice of priors and Markov chain Monte Carlo settings
☒	☐	For hierarchical and complex designs, identification of the appropriate level for tests and full reporting of outcomes
☒	☐	Estimates of effect sizes (e.g. Cohen's *d*, Pearson's *r*), indicating how they were calculated

Our web collection on statistics for biologists contains articles on many of the points above.

Software and code

Policy information about availability of computer code

Data collection	Confocal Images were captured with an A1RHD25 confocal microscope (Nikon imaging center, Curie Institut, Paris); Lattice Light Sheet Microscopy data were recorded with commercial LLSM 40 of 3i (Denver, USA) using two sCMOS cameras (Orca-Flash 4.0; Hamamatsu, Bridgewater, NJ); Electron microscopy negative staining images were acquired with Transmission electron microscope (TEM) 80 kV (Tecnai Spirit, ThermoFisher, USA), equipped with QUEMESA camera (Olympus) or TEM 80 kV (TEM 900, Zeiss) equipped with a Morada G2 camera (Olympus); CryoEM data and images were acquired with TEM 120 kV (Talos L120C, ThermoFisher), equipped with Ceta16M camera (ThermoFisher), TEM 300 kV (Titan Krios, ThermoFisher), equipped with K3 direct electron detector with energy filter (Gatan) and TEM 300 kV (Titan Krios, ThermoFisher), equipped with Falcon3 direct electron detector (ThermoFisher); Immunoblotting data were collected with BioRad ChemiDoc; FLIM and correlation microscopy data were collected with Microtime 200 system (PicoQuant GmbH, Germany); CHI760e Electrochemical Workstation (CH Instruments, USA) for electrochemical impedance spectroscopy; custom built TIRF Microscope (with 100 x objective, 1.45 NA) equipped with an ORCA-Flash4.0 V3 Digital CMOS camera (Hamamatsu) for photobleaching experiments; 121nanoAcquity UPLC device (WatersCorporation, Milford, MA, USA) and Q-Exactive HF-X mass spectrometer (ThermoFisher Scientific, Waltham, MA, USA) were used to generate cross-link proteomic data.
Data analysis	Confocal microscopy images were processed using ImageJ/Fiji softwares (version up to 2.9.0) as indicated. For co-localization analysis, the JACoP plugin run in ImageJ was used to measure Manders' coefficient signal co-occurrences.<br>For LLSM, automated tracking was calculated using the u-track software package, as part of cmeAnalysis3D software, which was implemented in Matlab 2021b; LLSM raw images, were deconvolved using LLSpy v0.4.8 (https://doi.org/10.5281/zenodo.3554482); LLSM Movies were rendered and visualized using Imaris software 9.82, or ImageJ/Fiji 1.53c; the analysis code can be found as part of the Github repository of llsmtools in https://github.com/francois-a/llsmtools/. Statistical analyses were performed using Prism v10.1.1 software (Graphpad Inc).<br>Photobleaching data were processed using Matlab-based open-source iSMS and AutoStepfinder software.<br>The MS/MS data were analyzed using MeroX software v2.0.1.4.

Electron microscopy Negative staining data were computed with crYOLO and cryoSPARC for particles autopicking and generation of 3D models respectively; for CryoElectron microscopy data, MotionCor2, cryoSPARC, Coot and ISOLDE software were used for particles alignement, 2D classification and atomic modeling.
Western blots were analyzed and quantified using Image Lab 6.1 (BioRad) and ImageJ/Fiji softwares (version up to 2.9.0).
Figures were all designed with Adobe Illustrator v29.4. Neither third party nor BioRender were used to build our schematics. Those are all designed by the corresponding author, Massiullah Shafaq-Zadah.

For manuscripts utilizing custom algorithms or software that are central to the research but not yet described in published literature, software must be made available to editors and reviewers. We strongly encourage code deposition in a community repository (e.g. GitHub). See the Nature Portfolio guidelines for submitting code & software for further information.

Data

Policy information about availability of data

All manuscripts must include a data availability statement. This statement should provide the following information, where applicable:

- Accession codes, unique identifiers, or web links for publicly available datasets
- A description of any restrictions on data availability
- For clinical datasets or third party data, please ensure that the statement adheres to our policy

All data supporting the findings of this study are available in the paper and its supplementary informations. CyroEM and cross-linking proteomic data are publicly available in a repository as detailed in the "Data availability" section of the manuscript

Research involving human participants, their data, or biological material

Policy information about studies with human participants or human data. See also policy information about sex, gender (identity/presentation), and sexual orientation and race, ethnicity and racism.

Reporting on sex and gender	*Use the terms sex (biological attribute) and gender (shaped by social and cultural circumstances) carefully in order to avoid confusing both terms. Indicate if findings apply to only one sex or gender; describe whether sex and gender were considered in study design; whether sex and/or gender was determined based on self-reporting or assigned and methods used. Provide in the source data disaggregated sex and gender data, where this information has been collected, and if consent has been obtained for sharing of individual-level data; provide overall numbers in this Reporting Summary. Please state if this information has not been collected. Report sex- and gender-based analyses where performed, justify reasons for lack of sex- and gender-based analysis.*
Reporting on race, ethnicity, or other socially relevant groupings	*Please specify the socially constructed or socially relevant categorization variable(s) used in your manuscript and explain why they were used. Please note that such variables should not be used as proxies for other socially constructed/relevant variables (for example, race or ethnicity should not be used as a proxy for socioeconomic status). Provide clear definitions of the relevant terms used, how they were provided (by the participants/respondents, the researchers, or third parties), and the method(s) used to classify people into the different categories (e.g. self-report, census or administrative data, social media data, etc.) Please provide details about how you controlled for confounding variables in your analyses.*
Population characteristics	*Describe the covariate-relevant population characteristics of the human research participants (e.g. age, genotypic information, past and current diagnosis and treatment categories). If you filled out the behavioural & social sciences study design questions and have nothing to add here, write "See above."*
Recruitment	*Describe how participants were recruited. Outline any potential self-selection bias or other biases that may be present and how these are likely to impact results.*
Ethics oversight	*Identify the organization(s) that approved the study protocol.*

Note that full information on the approval of the study protocol must also be provided in the manuscript.

Field-specific reporting

Please select the one below that is the best fit for your research. If you are not sure, read the appropriate sections before making your selection.

☒ Life sciences ☐ Behavioural & social sciences ☐ Ecological, evolutionary & environmental sciences

For a reference copy of the document with all sections, see nature.com/documents/nr-reporting-summary-flat.pdf

Life sciences study design

All studies must disclose on these points even when the disclosure is negative.

Sample size	Sample size for each experiment is indicated in figure captions. No statistical method was used to determine sample size prior to experiment.
Data exclusions	No data was excluded from statistical analysis.

Replication	Unless stated otherwise in the figure legends, all representative results shown for the different microspy, biophysical and biochemical approaches, and western-immunoblotting experiments were performed at least three times independently with similar results.
Randomization	For comparison between experimental groups (control vs treatment), the samples were asigned randomly to each group. All the control and treated experiments were done on the same day side by side using the same reagents batches.
Blinding	Investigators were not blinded as same person both collected and analysed the data.

Reporting for specific materials, systems and methods

We require information from authors about some types of materials, experimental systems and methods used in many studies. Here, indicate whether each material, system or method listed is relevant to your study. If you are not sure if a list item applies to your research, read the appropriate section before selecting a response.

Materials & experimental systems

n/a	Involved in the study
☐	☒ Antibodies
☐	☒ Eukaryotic cell lines
☒	☐ Palaeontology and archaeology
☒	☐ Animals and other organisms
☒	☐ Clinical data
☒	☐ Dual use research of concern
☒	☐ Plants

Methods

n/a	Involved in the study
☒	☐ ChIP-seq
☒	☐ Flow cytometry
☒	☐ MRI-based neuroimaging

Antibodies

Antibodies used

The following primary antibodies were used: hamster anti-a5 and b1 integrin primary antibody (BioLegend, Cat. # 103902, and 102202; 1:1000 for WB) ; mouse anti-α-tubulin (Sigma-Aldrich, clone B512, Cat. #T5168, 1:1000 for WB); rat mAb13 anti-b1 antibody (BD
20 Bioscience, Cat. #552828; 5 or 10 ug/ml for antibody binding and/or uptake assay), rat 9EG7 anti-b1 antibody (BD Bioscience, Cat. #553715; 10 ug/ml for antibody binding and/or uptake assay), mAb16 and SNAKA51anti-a5 antibodies (kindly provided by Patrick Caswe; 10 ug/ml for antibody binding and/or uptake assay), rabbit anti- SNAP-tag antibody (New England Biolabs, Cat. #P9310S; 1:1000 for WB), mouse anti-clathrin heavy chain antibody (BD Bioscience, Cat. #610500; 1:1000 for WB), mouse anti-vinculin antibody (Sigma-Merck, Cat. #V9131; 1:100 for IF), rabbit anti-vps35 antibody (kind gift from Juan Bonifacino; 1:1000 for WB), rabbit anti-vps26 antibody (Abcam, Cat.#ab23892; 1:1000 for IF);

The following secondary antibodies were used: secondary anti-mouse-HRP (Beckman Coulter, Cat. #715-035-151; 1:1000), secondary anti- rabbit-HRP (Beckman Coulter, Cat. #711-035 -152; 1:1000 for WB), secondary anti-rat-HRP (Beckman Coulter, Cat. #712-035-153; 1:1000 for WB), secondary anti-hamster-HRP (Beckman Coulter, cat. # 127-035-099; 1:1000 for WB), secondary anti-rat Cy3 (Beckman Coulter, Cat. #712-166-153; 1:200 for IF), secondary anti-mouse Alexa488 (Thermofisher, Cat. # ; 1:200 for IF)

Validation

The following primary antibodies have been characterized by their manufacturer:

Hamster anti-a5 integrin primary antibody:https://www.biolegend.com/fr-fr/products/purified-anti-mouse-rat-cd49e-antibody-304
Hamster anti-b1 integrin antibody: https://www.biolegend.com/fr-fr/products/purified-anti-mouse-rat-cd29-antibody-306
Rat anti-b1 integrin: https://www.bdbiosciences.com/en-au/products/reagents/flow-cytometry-reagents/research-reagents/single-color-antibodies-ruo/purified-rat-anti-human-cd29.552828?tab=product_details
Rat anti-b1 integrin: https://www.bdbiosciences.com/en-au/products/reagents/flow-cytometry-reagents/research-reagents/single-color-antibodies-ruo/purified-rat-anti-mouse-cd29.553715?tab=product_details
Mouse anti-α-tubulin: https://www.sigmaaldrich.com/FR/fr/product/sigma/t5168
Mouse anti-CHC:https://www.bdbiosciences.com/en-ca/products/reagents/microscopy-imaging-reagents/immunofluorescence-reagents/purified-mouse-anti-clathrin-heavy-chain.610500027
Rabbit anti-SNAP: https://www.neb.com/en/products/p9310-anti-snap-tag-antibody-polyclonal
Rabbit anti-VPS26: https://www.abcam.com/en-us/products/primary-antibodies/vps26-antibody-ab23892
Mouse anti-vinculin: https://www.sigmaaldrich.com/FR/fr/product/sigma/v9131

Other primary antibodies were validated "in house".

The secondary antibodies have been characterized by their manufacturer:
Donkey anti-mouse-HRP: https://www.jacksonimmuno.com/catalog/products/715-035-151
Donkey anti-rabbit-HRP: https://www.jacksonimmuno.com/catalog/products/711-035-152
Donkey anti-rat-HRP: https://www.jacksonimmuno.com/catalog/products/712-035-153
Goat anti-armenian hamster-HRP: https://www.jacksonimmuno.com/catalog/products/127-035-099
Goat anti-rat-Cy3: https://www.jacksonimmuno.com/catalog/products/712-166-153
Donkey anti-mouse-Alexa488: https://www.thermofisher.com/antibody/product/Donkey-anti-Mouse-IgG-H-L-Highly-Cross-adsorbed-Secondary-Antibody-Polyclonal/A-21202

Eukaryotic cell lines

Policy information about cell lines and Sex and Gender in Research

Cell line source(s)	HeLa; HeLa stably expressing TGN GalT-GFP-SNAP fusion protein; RPE-1; genome edited RPE-1 cells stably expressing AP2-mTag-GFP; a5b1 integrin double KO mouse kidney fibroblast (MKF-dKO, kindly provided by Reinhard Fässler)
Authentication	None used.
Mycoplasma contamination	All cell lines were regularly tested for mycoplasma contamination either in our lab (Mycoalert PLUS detection kit, #LT07-710; Lonza) or by the GATC/eurofins company (Mycocheck service ; https://eurofinsgenomics.eu/).
Commonly misidentified lines (See ICLAC register)	No commonly misidentified cell lines were used in this study.

Plants

Seed stocks	*Report on the source of all seed stocks or other plant material used. If applicable, state the seed stock centre and catalogue number. If plant specimens were collected from the field, describe the collection location, date and sampling procedures.*
Novel plant genotypes	*Describe the methods by which all novel plant genotypes were produced. This includes those generated by transgenic approaches, gene editing, chemical/radiation-based mutagenesis and hybridization. For transgenic lines, describe the transformation method, the number of independent lines analyzed and the generation upon which experiments were performed. For gene-edited lines, describe the editor used, the endogenous sequence targeted for editing, the targeting guide RNA sequence (if applicable) and how the editor was applied.*
Authentication	*Describe any authentication procedures for each seed stock used or novel genotype generated. Describe any experiments used to assess the effect of a mutation and, where applicable, how potential secondary effects (e.g. second site T-DNA insertions, mosiacism, off-target gene editing) were examined.*
